# Stretchable plasmonic metasurfaces for deformation monitoring

**DOI:** 10.1515/nanoph-2024-0461

**Published:** 2024-10-16

**Authors:** Peiyang Li, Kaikai Gao, Ruize Ma, Kai Pan, Dong Li, Feng Liu, Peng Li, Xuetao Gan, Jianlin Zhao, Dandan Wen

**Affiliations:** Key Laboratory of Light Field Manipulation and Information Acquisition, Ministry of Industry and Information Technology, and Shaanxi Key Laboratory of Optical Information Technology, School of Physical Science and Technology, 26487 Northwestern Polytechnical University, Xi’an 710129, China

**Keywords:** metasurface, Hologram, flexible material, stretchable electronics, optical sensor

## Abstract

Metasurfaces have recently gained significant attention due to the strong capacity in light field manipulation. However, most traditional metasurfaces are fabricated on rigid substrates, which fix their functionality after fabrication and limit their applications in dynamic measurement fields. In this work, we designed and fabricated a silver metasurface embedded in a stretchable substrate for sensing applications. This metasurface can generate different point cloud patterns under varying stretch ratios when illuminated by a laser beam. By collecting and analyzing the patterns, we can precisely reconstruct the deformation of the metasurface. Furthermore, the sample exhibits excellent performance under incident light of various wavelengths. These results pave the way for developing microdevices with novel capabilities based on flexible metamaterials.

## 1 Introduction

Optical metasurfaces, as two-dimensional subwavelength devices [1], have emerged as a powerful platform for manipulating incident light at the nanoscale. They can effectively control the amplitude [2], phase [2], [3], [4], and polarization [5], [6], [7] of light, bringing new possibilities to various fields. For instance, applications such as metasurface holography [8], [9], [10], [11], [12], [13], [14], [15], spectral reconstruction [16], [17], generation of special light field [18], [19], [20], and optical information encryption [21], [22], [23] have been successfully demonstrated. However, most traditional metasurfaces are fabricated on rigid substrates such as silicon dioxide, and their functions are usually fixed after fabrication. Additionally, the nanostructures of most traditional metasurfaces are exposed to the air, making them prone to rapid and permanent failure in non-cleanroom environments. This not only increases maintenance costs but also limits the prospects for large-scale applications of metasurfaces. Therefore, enhancing the stability and flexibility of metasurfaces has become a crucial research focus.

In recent years, flexible substrate-based metasurfaces have garnered significant attention. For instance, the focal length of metalenses can be dynamically adjusted by altering the shape of the flexible substrate [24], [25]. Tunable structural colors can be achieved by adjusting the deformation of the flexible substrate to change the relative positions of the nanostructures, thereby altering the perceived color [26], [27], [28], [29]. Additionally, phase compensation devices can use the substrate’s deformation to correct phase distortions in the wavefront [30], [31]. In addition to light field modulation purposes, flexible substrate-based metasurfaces also introduce greater flexibility and innovation to sensor design. For example, integrating sensors on flexible metasurfaces can enable real-time monitoring and control of environmental parameters such as stress [32]. However, previous work has primarily relied on measuring different transmission and reflection spectra under deformation conditions, establishing a correlation between deformation and spectra for sensing purposes [32], [33], [34]. Although this method has been successfully demonstrated, the complexity of spectral optical systems limits the further application of flexible metasurface sensing.

In this study, we proposed and experimentally validated a flexible geometric metasurface for sensing application. The nanostructures are encapsulated between two layers of flexible material, safeguarding them from air exposure and enhancing durability and stability. This double-layer structure also offers superior mechanical support, ensuring robust functionality under stress. Illuminated by incident light, the metasurface directly forms point cloud patterns on the observation plane, with different patterns under varying stretching conditions captured using a CCD to infer the flexible material’s deformation. This metasurface exhibits broadband characteristics because the phase delay depends solely on the orientation of the nanorods, enabling effective operation across multiple wavelength ranges. This broadband capability enhances the metasurface’s flexibility and practicality in various applications. The method facilitates high-precision deformation measurements of flexible materials through straightforward image analysis, eliminating the need for complex spectral measurement systems. This approach opens new avenues for sensing applications of flexible metasurfaces and promises broad practical implications.

## 2 Results

The Dammann grating (DG) is a type of grating element based on binary optical technology [35]. It is used to split an incident light beam into multiple diffraction orders at equal intervals, creating a uniform light intensity distribution across these orders. The transmission function *T*(*x*) of a DG can be described using an infinite series via Fourier expansion:

(1) $$T\left(x\right)=\exp\left[\text{i}\varphi_{DG}\left(x\right)\right]=\overset{+\infty }{\underset{n=-\infty }{\sum }}C_{n}\exp\left(\text{i}2\pi xn/\Lambda \right)$$

where, *φ*_DG_ represents the phase function of the DG, Λ is the period of the DG, and *C*_*n*_ are the coefficients of each term in the infinite series after Fourier expansion. The expression for *C*_*n*_ is

(2) $$C_{n}=\left\{\begin{array}{cc} \frac{-\text{i}}{2n\pi }\left[1+2\overset{N-1}{\underset{k=1}{\sum }}{\left(-1\right)}^{k}\exp\left(-i2\pi nx_{k}\right)+{\left(-1\right)}^{N}\exp\left(-i2\pi nx_{N}\right)\right]\; & n\neq 0\\ 2\overset{N-1}{\underset{k=1}{\sum }}{\left(-1\right)}^{k}x_{k}+{\left(-1\right)}^{N}x_{N}\; & n=0 \end{array}\right.$$

where, *x*_*k*_ denotes the normalized phase transition points within each period, with *x*_0_ = 0 and *x*_*N*_ = 1, *N* is the total number of transition points, and |*C*_*n*_|^2^ represents the power of the *n*th order, normalized relative to the total power. As shown in Eq. (2), *C*_*n*_ is independent of the grating constant Λ and only depends on the total number *N* of transition points and the spatial coordinates *x*_*k*_. Optimizing the values and number of *x*_*k*_ allows efficient and uniform distribution of incident light into the desired *M* diffraction orders. The grating constant Λ determines the distance between adjacent diffraction orders in the Fourier plane, thereby affecting the beam’s divergence angle.

**Figure 1:**
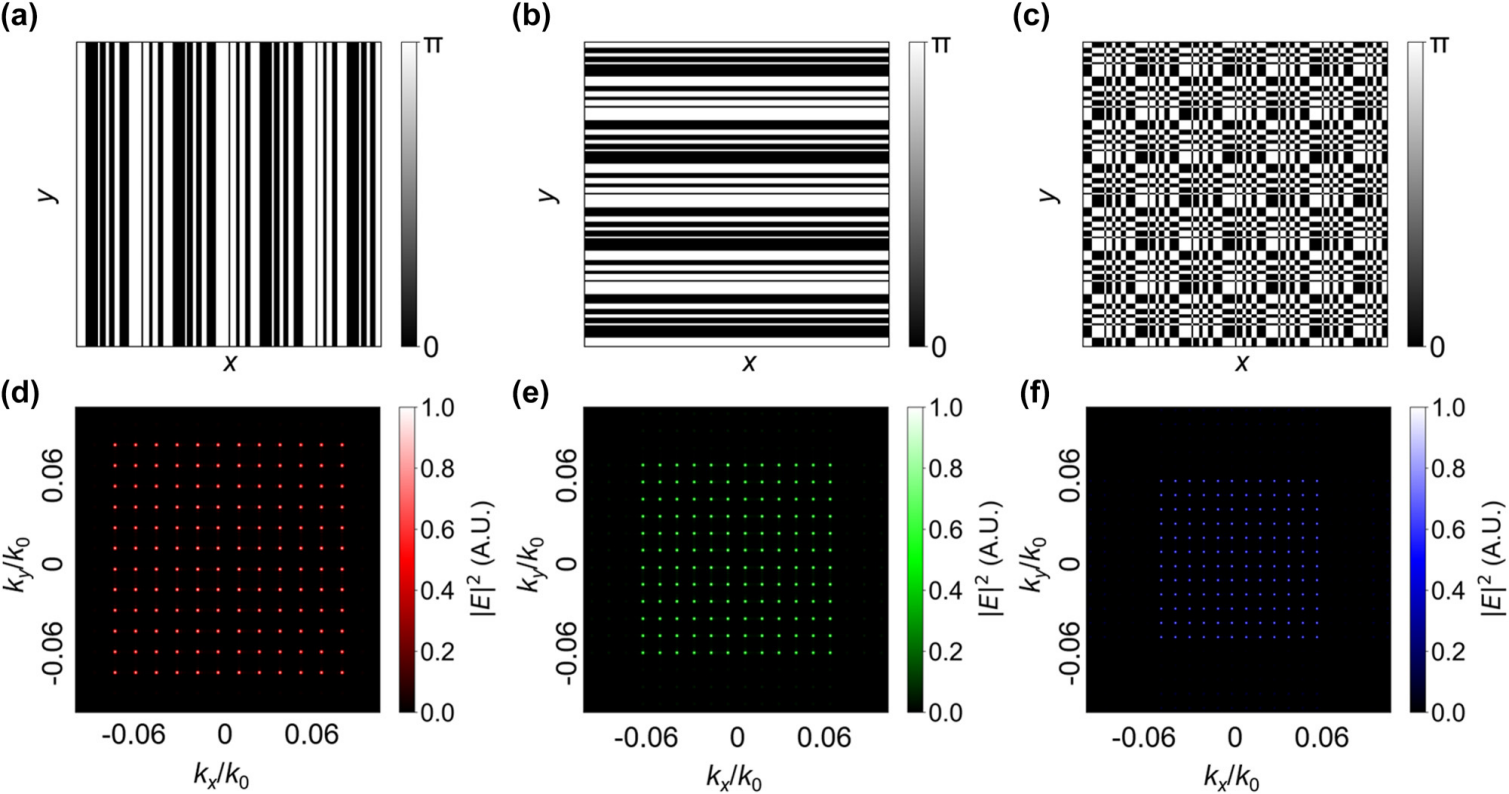
Phase distribution and far-field intensity of DGs (a) and (b) Schematic of the phase distribution of 1D DGs along the *x*-axis and *y*-axis. (c) Phase distribution of a 2D DG, which is the superposition of the 1D DG phases from (a) and (b). Far-field intensity distribution of 12 × 12 diffraction orders generated with a 2D DG. The period of DG is 100 μm, and the distance between the DG and observation plane is 20 cm. Here we use normalized wave vector coordinates *k*_*x*_/*k*_0_ and *k*_*y*_/*k*_0_ as *x*-axis. The incident wavelengths are (d) 633 nm, (e) 532 nm, and (f) 450 nm.

Figure 1(a) illustrates the spatial distribution of the phase of a 1D DG with a grating constant Λ of 100 μm and 12 diffraction orders along the *x*-axis, while Figure 1(b) shows the distribution along the *y*-axis. The phase distribution of a 2D DG is derived by combining the phase distributions of two orthogonal 1D DG using a modified **XOR** operation [36] (the output is 0 or π if the two values are the same or not, respectively) [Figure 1(c)]. Next, the calculated transmission function of the 2D DG is used as the aperture plane transmission distribution. The observation plane is far from the aperture plane, well beyond the Fresnel diffraction region. Therefore, the field in the observation plane [*U*(*x*, *y*)] can be found directly from a Fourier transform of the aperture plane [*U*(*x*_0_, *y*_0_)]:

(3) $$\begin{array}{ll} U\left(x,y\right) & =\frac{1}{\text{j}\lambda z}\exp\left(\text{j}kz\right)\exp\left[\text{j}\frac{k}{2z}\left(x^{2}+y^{2}\right)\right]\\ & \;\times F\left\{U\left(x_{0},y_{0}\right)\right\} \end{array}$$

where, *λ* represents the wavelength of incident light, *k* represents the wave number, and *z* represents the propagation distance. According to the holographic diffraction formula, the size of the real image point is

(4) $$\Delta x_{obser}=\frac{\lambda z_{0}}{D_{x}}$$

where, *D*_*x*_ is the extent of the aperture plane. It can be seen that when the propagation distance *z*_0_ is fixed, ∆*x*_obser_ is directly proportional to the wavelength *λ*. It can be seen from Eq. (1) that the diffraction angles of different orders are determined by Λ. Here Λ is fixed at 100 μm. When the number of diffraction orders is rather limited, the measurement points for inferring deformation based on spots displacement may be insufficient, and the intensity distribution of the spots array in the far field may be covered by the central zero-order spot (Supplementary Figure 1). The diffraction patterns show the normalized wave vector directions in the *x* and *y* directions (*k*_*x*_/*k*_0_), and the diffraction angle can be expressed as *θ* = arcsin(*k*_*x*_/*k*_0_). Imaging distortion and measurement inaccuracies occur when there are too many diffraction orders, as the imaging angle *θ* of the edge orders in the far field becomes too large, violating the paraxial approximation condition. Here, a moderate number of diffraction orders, specifically 12 × 12, are used to ensure an adequate number of diffraction orders for measurement purposes. Assuming an incident light wavelength of 633 nm, the maximum diffraction angle calculated under these conditions is approximately 5.6°, which can be considered to satisfy the paraxial approximation condition. These calculations indicate that the diffraction orders obtained under different incident wavelengths will also exhibit different diffraction angles. Figure 1(d)–(f) illustrate the calculated intensity distribution on the observation plane for incident wavelengths of 633 nm, 532 nm and 450 nm, respectively. It can be estimated that the maximum diffraction angles are approximately 5.6°, 4.7° and 4.0°, respectively.

**Figure 2:**
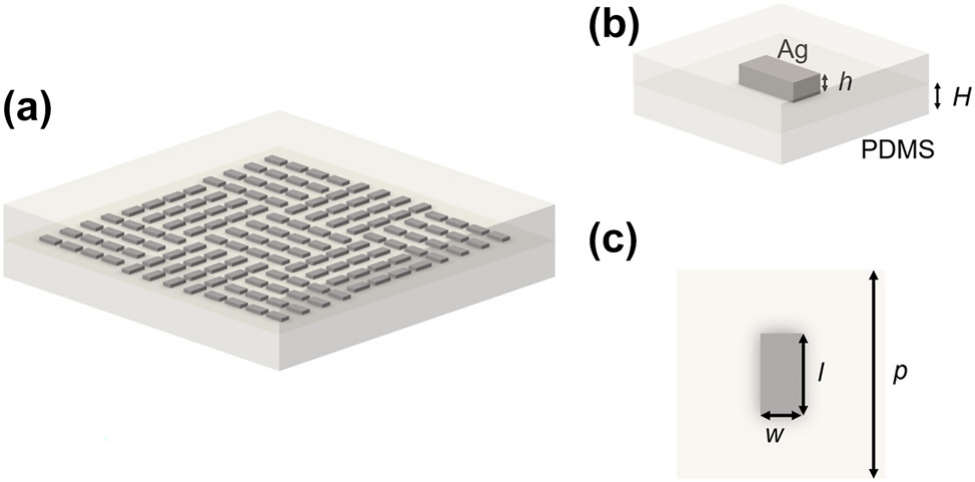
Metasurface encapsulated within a flexible material. (a) Schematic of a 2D flexible DG metasurface, with silver nanorods encapsulated in upper and lower PDMS layers, each with a height of *H* ≈ 0.5 mm. (b) Schematic of the structure within a single period *p* = 350 nm, showing silver nanorods with a length *l* = 200 nm, width *w* = 100 nm, and height *h* = 30 nm. (c) Top view of the unit cell structure.

Next, we encode the obtained 2D DG’s phase distribution information onto the geometric metasurface, which achieves different phase delays by altering the rotation angle of individual nanostructure. The structure of the metasurface is depicted in Figure 2(a). The used DG belongs to binary phase devices, thus the rotation angle of the nanostructures is either 0° or 90°. Figure 2(b) and (c) illustrate a schematic of the structure within a single period. The silver nanorods have the same length of 200 nm, width of 100 nm, and height of 30 nm. The initial spacing between adjacent nanorods is 350 nm. These nanorods are encapsulated within upper and lower Polydimethylsiloxane (PDMS) layers, each with a height of 0.5 mm. Unlike metasurface with single-layer PDMS substrate, this kind of sample effectively prevents the detachment of silver nanorods during tensile deformation, while the dual-layer PDMS also protects the nanostructures from dust contamination and prevents the silver nanorods from oxidation. In this study, since the size of the metal structure region of the metasurface is 350 μm × 350 μm, and the entire PDMS is 3 cm × 1 cm, much larger than the metasurface area, it can be assumed that the deformation induced by stress in the sample is uniform (Supplementary Figure 2).

**Figure 3:**
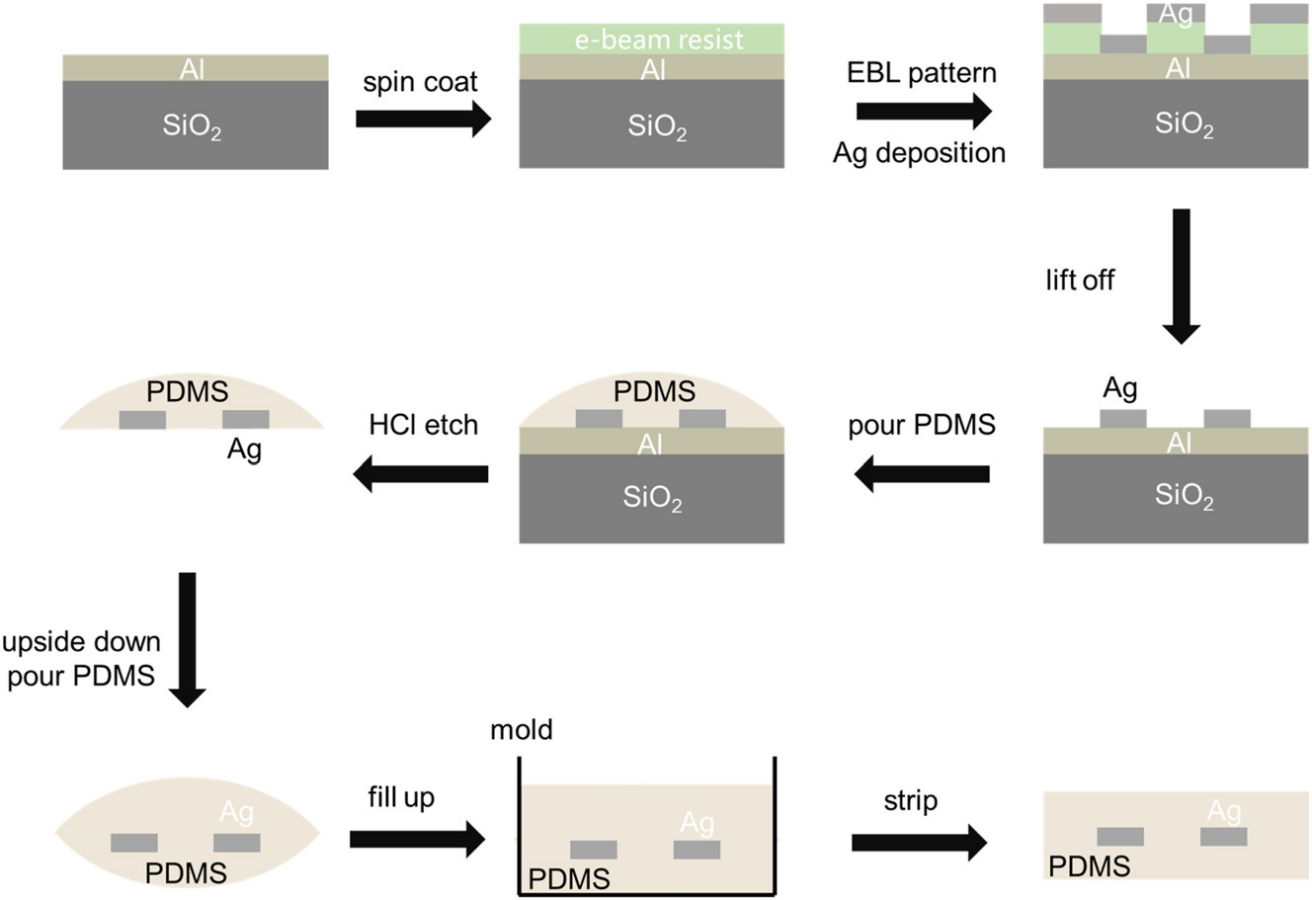
Fabrication process of the flexible metasurface.

The fabrication process of the flexible substrate metasurface is shown in Figure 3. First, a 30 nm thick aluminum film is deposited on a silicon dioxide substrate by thermal evaporation at a pressure of 5 × 10^−4^ Pa. Next, a layer of 200 nm thick positive electron-beam resist (AR-P 6200.13) is spin-coated onto the aluminum film. Patterns are then defined using electron-beam lithography, followed by development. Subsequently, a 30 nm thick silver film is deposited by thermal evaporation at a pressure of 5 × 10^−4^ Pa. Afterwards, the sample is immersed in N-methyl-2-pyrrolidone (NMP) solution at room temperature for 1 h to dissolve excess resist. Approximately 0.5 mm thick PDMS (with a ratio of 10:1 of PDMS base to curing agent) is poured onto the substrate and baked at 80 °C for 2 h. The sample is then left at room temperature for 24 h to enhance the adhesion between PDMS and silver nanorods. In the next, the sample is immersed in a 6 mol/L HCl solution for 10 min to dissolve the aluminum film, transferring the silver nanostructures onto the PDMS substrate. Another PDMS film is prepared on the other side using the same method, encapsulating the silver nanorods within PDMS. Due to the high surface tension of PDMS, liquid at the edges converges towards the center during PDMS curing, resulting in a large curvature at the center of the final sample. The entire dual-layer PDMS acts like a converging lens, causing a smaller maximum diffraction angle measured in experiments. To address this issue, a silicon cuboid mold is fabricated, and the entire sample is placed into the mold after transferring the metasurface array into the dual-layer PDMS. The inner surface of the mold is treated with a polytetrafluoroethylene (PTFE) solution to form a molecular layer, preventing the cured PDMS from tightly adhering to the glass mold. PDMS is then poured into the mold and baked at 80 °C for 2 h. Finally, the sample is removed from the cuboid mold, resulting in a flat-surfaced sample. The schematic diagram of the sample is shown in Supplementary Figure 3.

**Figure 4:**
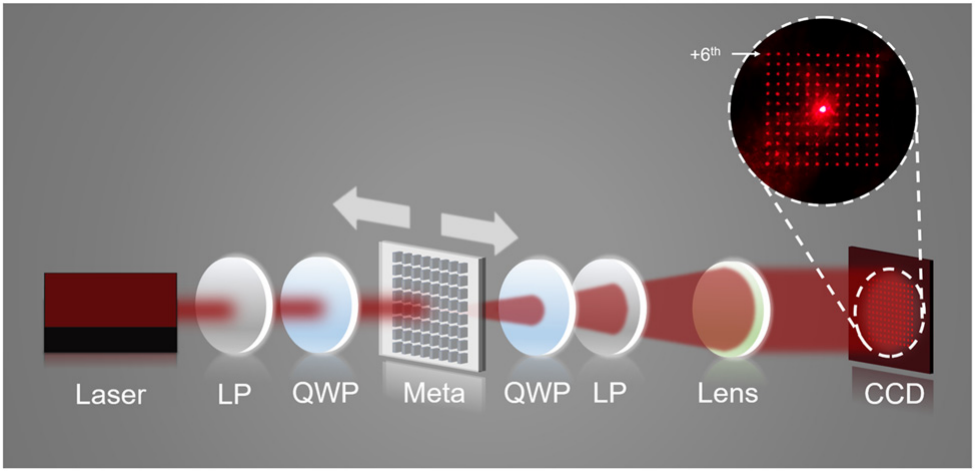
Schematic of the experimental setup. LP, linear polarizer; QWP, quarter-wave plate; MS, metasurface.

The experimental setup is illustrated in Figure 4. A laser beam passes through a polarizer, it then goes through a quarter-wave plate to convert into circularly polarized. After interacting with the metasurface, the transmitted circularly polarized light with opposite helicity was selected with another pair of quarter-wave plate and polarizer. Finally, the light is imaged onto a CCD camera through a lens, with an exposure time of 0.4 ms. The distance between the CCD and the lens is 35 mm. It is worth noting that collecting transmitted light may present some technical challenges in certain practical deformation monitoring applications. However, our sample design responds to both transmitted and reflected light, with the signal-to-noise ratio measured by the CCD in the reflection-type optical setup being comparable to that in the transmission-type optical setup. The reflection-type optical setup can be easily realized by placing a beam splitter 2 cm in front of the sample, the reflected light from the sample is filtered by a quarter-wave plate and a linear polarizer, collected by a lens and captured by the CCD (please refer to Supplementary Figure 6 for the reflected cloud pattern image). The subsequent measurements in this paper are based on the results obtained from the transmission-type optical setup.

**Figure 5:**
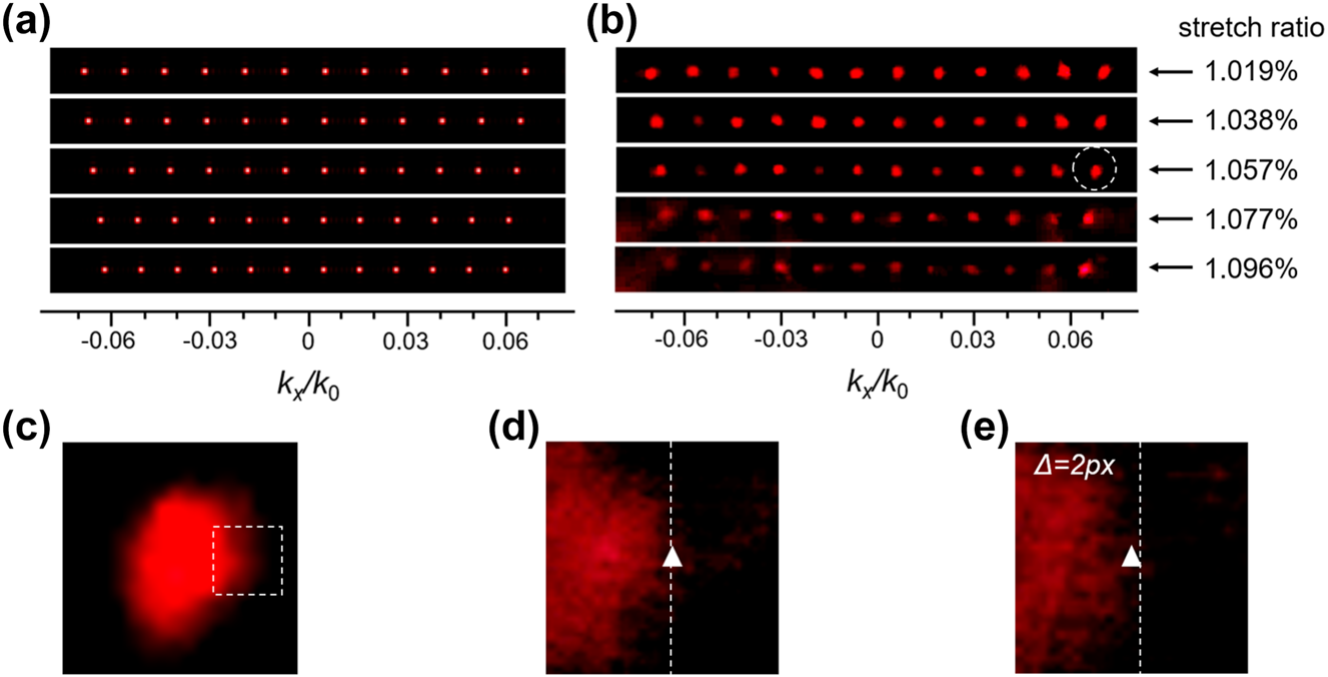
Schematic diagrams of the far-field spots obtained from theoretical calculations (a) and experimental measurements (b) under different strain ratios. The spots are taken from the diffraction orders in the +6th row along the *y*-axis of the 12 × 12 lattice (as shown in Figure 4). (c) The spot in the white dashed circular frame in (b) under a strain ratio of 1.057 %. (d) An enlarged view of the spot in the white dashed square frame in (c), with white triangle symbols indicating the position of the rightmost pixel selected in the actual measurement. (e) A schematic diagram of the spot in (d) under a slightly increased strain. The white dashed lines in (d) and (e) remain in the same position, and the white triangle symbols in both figures are 2 pixels apart horizontally.

During measurements, the sample is first clamped between two optical mounts, where the distance between the metasurface region and the two mounts are the same. This ensures that when equal displacements occur on both sides, the center of the diffracted patterns (i.e., the zeroth order) remains in the same position. In the experiments, the optical mounts are fixed on a three-axis translation stage, and the displacement of the mounts is precisely controlled by a screw micrometer. As PDMS is composed of cross-linked long-chain molecules, these molecules tend to be randomly arranged in their natural state. Under stress, these long-chain molecules align along the stress direction, causing the entire sample to exhibit macroscopic anisotropy. Therefore, it is necessary to adjust the quarter-wave plate behind the metasurface in a timely manner to maximize the transmitted circularly polarized light with opposite helicity on the observation screen when stretching the sample. It is worth noting that the 2D grating has a C_4_ symmetry structure, and stretching in the *y*-direction will yield the same results as in the *x*-direction. In our experiments, due to the PDMS being processed into a rectangular shape (please find in the Supplementary Figure 3), stretching in other directions (such as 45°) may not lead to uniform deformation of the sample. This could affect our measurements and analysis of deformations along that direction. Therefore, this paper chooses stretching in the *x*-direction as the measurement target. Figure 5(a) illustrates the deformation of the light spots under different stretching ratios obtained from numerical calculations. The spacing *p* between the metasurface nanorods increases linearly with stretching of the flexible metasurface. According to Eq. (4), it can be observed that, with the wavelength *λ* and propagation distance *z*_0_ fixed, the extent of the metasurface is inversely proportional to the size of the real image point on the observation plane. In other words, as the sample stretches, the image formed on the observation plane will be proportionally compressed. Assuming an incident light wavelength of 633 nm and a propagation distance of *z*_0_ = 20 cm, the width of the diffraction orders on the observation plane calculated using Eq. (3) is 27 mm in the unstretched state of the sample, whereas the experimental data is nearly consistent with theory at 25 mm.

When calculating the spacing between diffraction orders collected by the CCD camera, we need to consider that the light spots on the CCD have a certain size rather than forming infinitely small dots. Additionally, the shapes of these light spots may slightly change during sample deformation, resulting in imperfect circular images for specific diffraction orders. Therefore, some post-processing of the collected images is necessary to determine the center position of these diffraction orders. The used processing method, as shown in Figure 5(b) and (c), involves normalizing the intensity of the captured images, selecting the first pixel on the right side of a specific diffraction order with an intensity lower than half the maximum intensity as the right boundary of the diffraction order. Here we mark its position with a white triangle symbol. Similarly, the left boundary of the diffraction order can be chosen, and the geometric mean of the left and right boundary positions is calculated to obtain the center of the diffraction order. In this work, ±5 and ±6 diffraction orders at the outermost periphery were selected during experimental measurements to avoid noise interference from the central zero-order light. Specifically, when a sample is subjected to stress along the *x*-direction, we first choose the order in the upper left corner (Figure 4), and measure its distance from the adjacent orders in the *x* and *y* directions, and mark as ∆*x*_1_ and ∆*y*_1_; Similarly, we choose the order in the upper right corner, measure its distance from the adjacent orders in the *x* and *y* directions, and mark as ∆*x*_2_ and ∆*y*_2_. In the same way, we get ∆*x*_3_ and ∆*y*_3_ from the bottom left corner, and ∆*x*_4_ and ∆*y*_4_ from the bottom right corner. Finally, this average spacing (∆*x*_1_ + ∆*x*_2_ + ∆*x*_3_ + ∆*x*_4_)/4 in the *x*-direction and (∆*y*_1_ + ∆*y*_2_ + ∆*y*_3_ + ∆*y*_4_)/4 in the *y*-direction is compared with the initial spacing to determine the deformation ratio of the sample. Figure 5(d) and (e) show the variation of the boundary pixel values of the diffraction orders collected on the CCD under slight stretching. The sensitive area of the CCD has 896 × 684 pixels, and the white dashed line represents the position of the right boundary pixel during the first measurement. In the experiment, the minimum detectable pixel change on the CCD is 2, corresponding to a minimum measurable stretching ratio of 0.0057 % at 633 nm.

**Figure 6:**
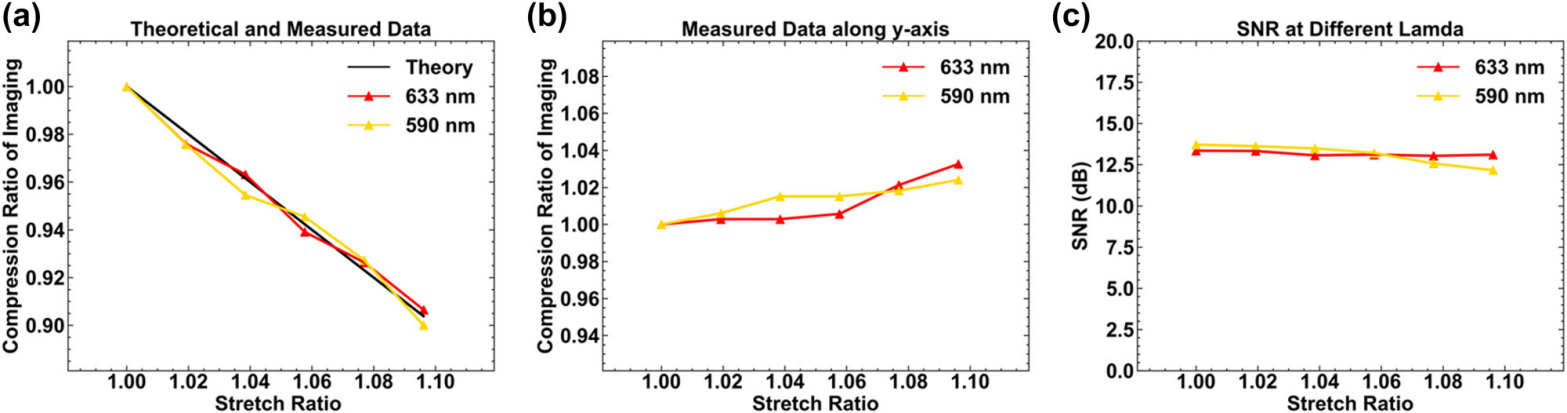
Experimentally measured stretch ratio and signal to noise ratio at 633 nm and 590 nm. (a) The black solid line represents the strain ratio derived from the micrometer screw, while the colored solid lines represent the strain ratio derived from the diffraction order displacement. (b) The strain ratio derived from the diffraction order displacement in the *y*-direction during the experiment. (c) The experimentally measured signal-to-noise ratio.

In our experiments, we measured the transmittance of PDMS in the visible to near-infrared range, and the results showed that its transmittance exceeds 95 % in this range (please refer to Supplementary Figure 5), indicating that PDMS remains highly transparent within this wavelength range. Therefore, the transparency of PDMS has a negligible impact on the experimental results. Figure 6(a) shows a comparison of the strain ratio derived from the micrometer screw (black solid line) with those derived from the diffraction order displacement (colored solid lines) under incident light of 633 nm and 590 nm. It can be seen that the experimental data fit well under the incident conditions of 633 nm and 590 nm. It is worth noting that when stretching PDMS along the *x*-direction, slight compression occurs in the *y*-direction perpendicular to the stretching direction, causing a slight expansion in the spacing between diffraction orders along the *y*-direction. Figure 6(b) illustrates the schematic of the deformation of the light spots along the *y*-direction perpendicular to the stretching direction along the *x*-direction, showing that the deformation in the *y*-direction is within 3 % when stretching along the *x*-direction is up to 10 %.

Signal-to-noise ratio is also an important parameter for evaluating the performance of the sample. We first used a power meter to measure the power of the diffraction orders used to calculate the strain ratio. Then, with the entire optical setup unchanged, two translation stages are moved to ensure that the incident light shines on the PDMS without the metasurface structure. The power of the scattered light caused by the PDMS material itself is measured as the background noise at this wavelength. To minimize the potential errors associated with individual measurements, we averaged the power of 16 diffraction orders in a single measurement, obtaining a signal intensity of 0.042 μW for a single diffraction order, with the background noise for an area of the same size being 0.002 μW. Substituting these values into the signal-to-noise ratio formula *SNR* = 10lg(*P*_s_/*P*_n_), where *P*_s_ is the signal power and *P*_n_ is the noise power, the initial signal-to-noise ratio is calculated to be 13.44 dB. The signal-to-noise ratio was measured under different stretching conditions, as shown in Figure 6(c). It can be observed that at an incident wavelength of 633 nm and 590 nm, the signal-to-noise ratio remains nearly unchanged under deformation conditions within 10 %. However, after approximately 11 % stretching, the PDMS scattering sharply increases, causing a rapid decline in the signal-to-noise ratio. Moreover, this speckle noise cannot be filtered out by adjusting the quarter-wave plate or the polarizer after the metasurface, resulting in diffraction order signals being submerged in the speckle noise, making it difficult to accurately locate the boundary pixels and central pixels of the diffraction order using pixel positioning methods. Therefore, the normal operating range of the sample at 633 nm and 590 nm is approximately 0 %–10 % deformation. We also measured the stretch ratio and SNR at 532 nm (please find in the Supplementary Figure 4 and Supplementary Note 1).

## 3 Conclusions

In this study, we introduced a straightforward method for fabricating a silver metasurface embedded in PDMS. Through far-field imaging and experimental measurements, we investigated the variations in imaging under different deformations. Remarkably, the deformation parameters inferred from imaging changes align closely with those experimentally measured results. This alignment underscores the efficacy of our approach and its ability to accurately capture deformations. Our method not only demonstrates the feasibility of fabricating metasurfaces on PDMS substrates but also highlights their potential for applications requiring precise control over deformations. Provided that the electron beam lithography could be replaced by the deep ultraviolet (DUV) photolithography [37], the device here is capable of large-scale production. Our work contributes to advancing the understanding and utilization of flexible metasurfaces in various fields, including optical sensing and imaging technologies.

## Supporting Information

Far-field intensity distribution diagram of a 5 × 5 array DG with a period of 100 μm; diagram of the DG under an optical microscope; illustration of the sample prepared for the experiment; experimentally measured stretch ratio and signal to noise ratio at 532 nm; detailed analysis of discrepancies at 532 nm.

## Supplementary Material

Supplementary Material Details
