# Supplementary material for: Stretchable plasmonic metasurfaces for deformation monitoring
Source: Nanophotonics. 2024 Oct 16;13(24):4483–90. doi: 10.1515/nanoph-2024-0461 (PMC11636405; doi:10.1515/nanoph-2024-0461)
Supplement: Supplementary file 1 — Supplementary Material Details [file j_nanoph-2024-0461_suppl_001.docx]

**Supporting Information for “Stretchable Plasmonic Metasurfaces for Deformation Monitoring”**

Peiyang Li, Kaikai Gao, Ruize Ma, Kai Pan, Dong Li*, Feng Liu, Peng Li, Xuetao Gan, Jianlin Zhao and Dandan Wen*

Key Laboratory of Light Field Manipulation and Information Acquisition, Ministry of Industry and Information Technology, and Shaanxi Key Laboratory of Optical Information Technology, School of Physical Science and Technology, Northwestern Polytechnical University, Xi’an 710129, China

**Corresponding authors:** [**dongli@nwpu.edu.cn**](mailto:dongli@nwpu.edu.cn)**;** [**dandanwen@nwpu.edu.cn**](mailto:dandanwen@nwpu.edu.cn)


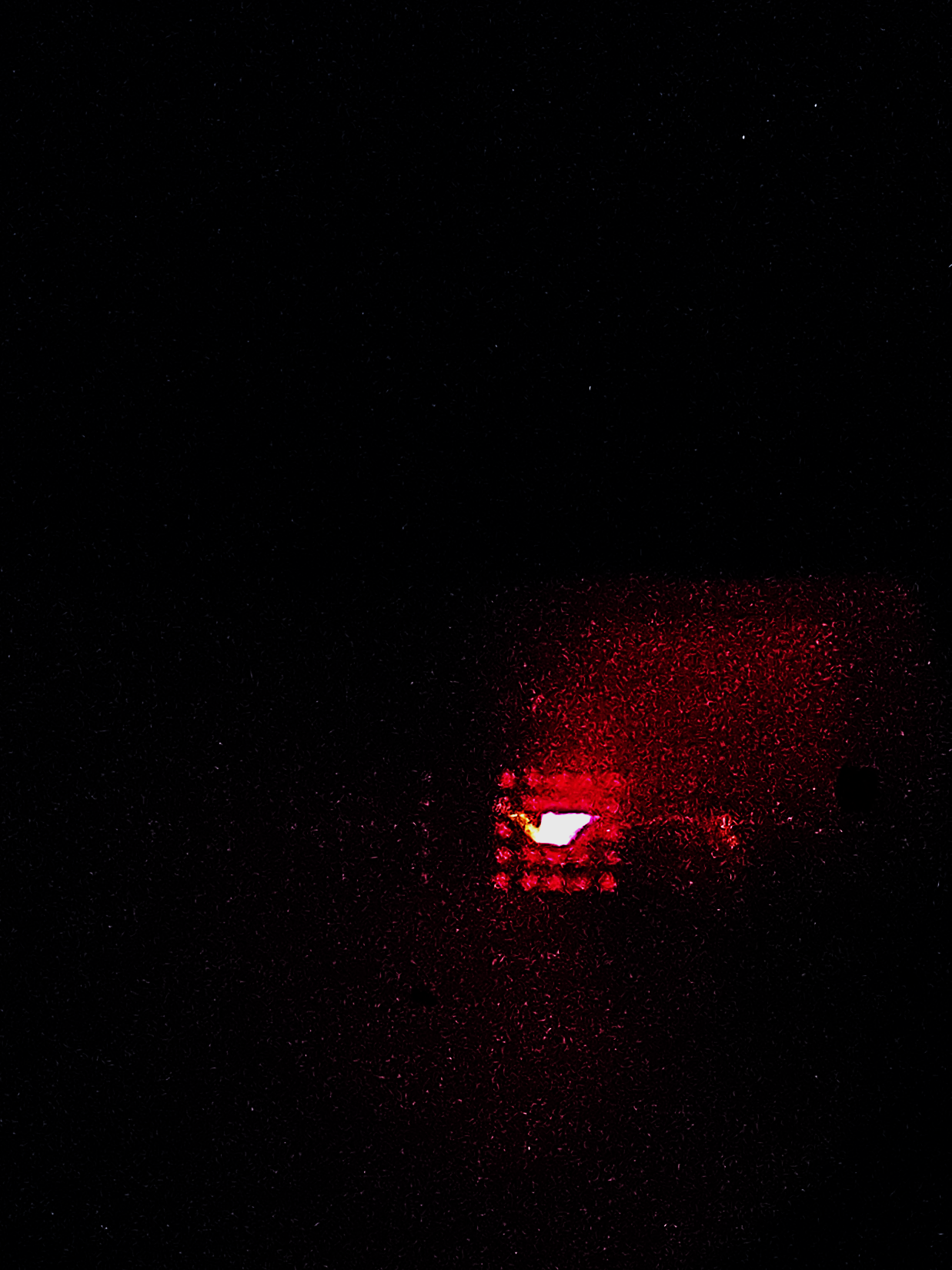


**Supplementary Figure 1**. Far-field intensity distribution diagram of a 5 × 5 array DG with a period of 100 μm. The spreading of the central zero-order spot overwhelms the ± 1 and ± 2 diffraction orders, resulting in a reduced number of usable measurement points.


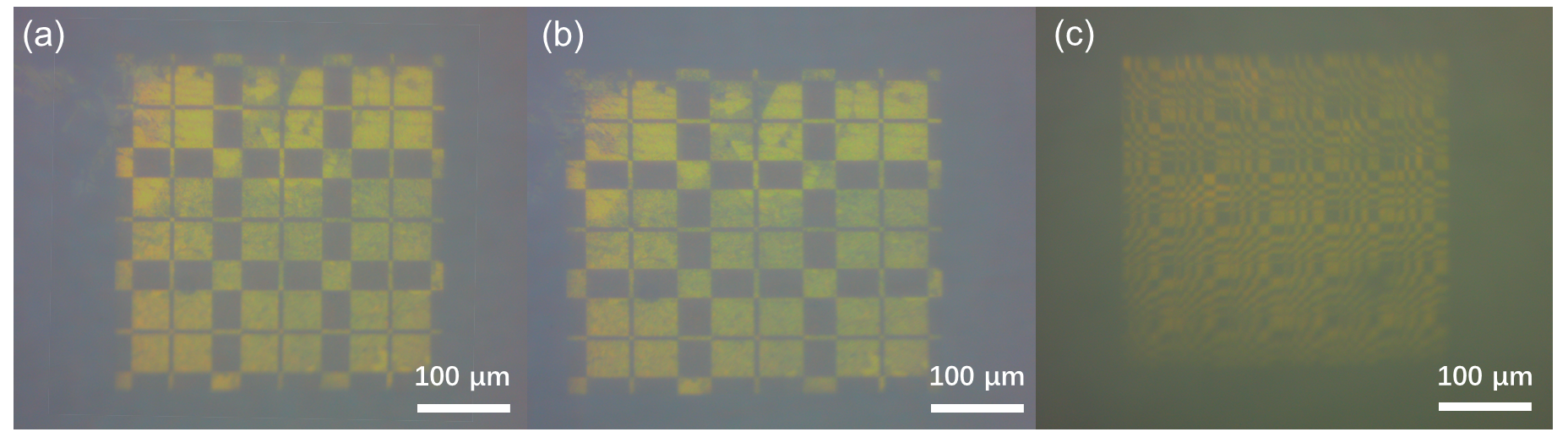


**Supplementary Figure 2**. Diagram of the DG under an optical microscope. (a) Reflective optical microscope image of a 5 × 5 DG with a period of 100 μm in an unstretched state. The ununiform intensity distribution in the microscopic image is due to e-beam lithography errors, where the areas with horizontal nanorods and vertical nanorods are slightly different in geometry. (b) Image of the structure in (a) under approximately 3% stretching, showing uniform deformation of the metasurface. (c) Diagram of the 12 × 12 DG with a period of 100 μm used in the experiment.


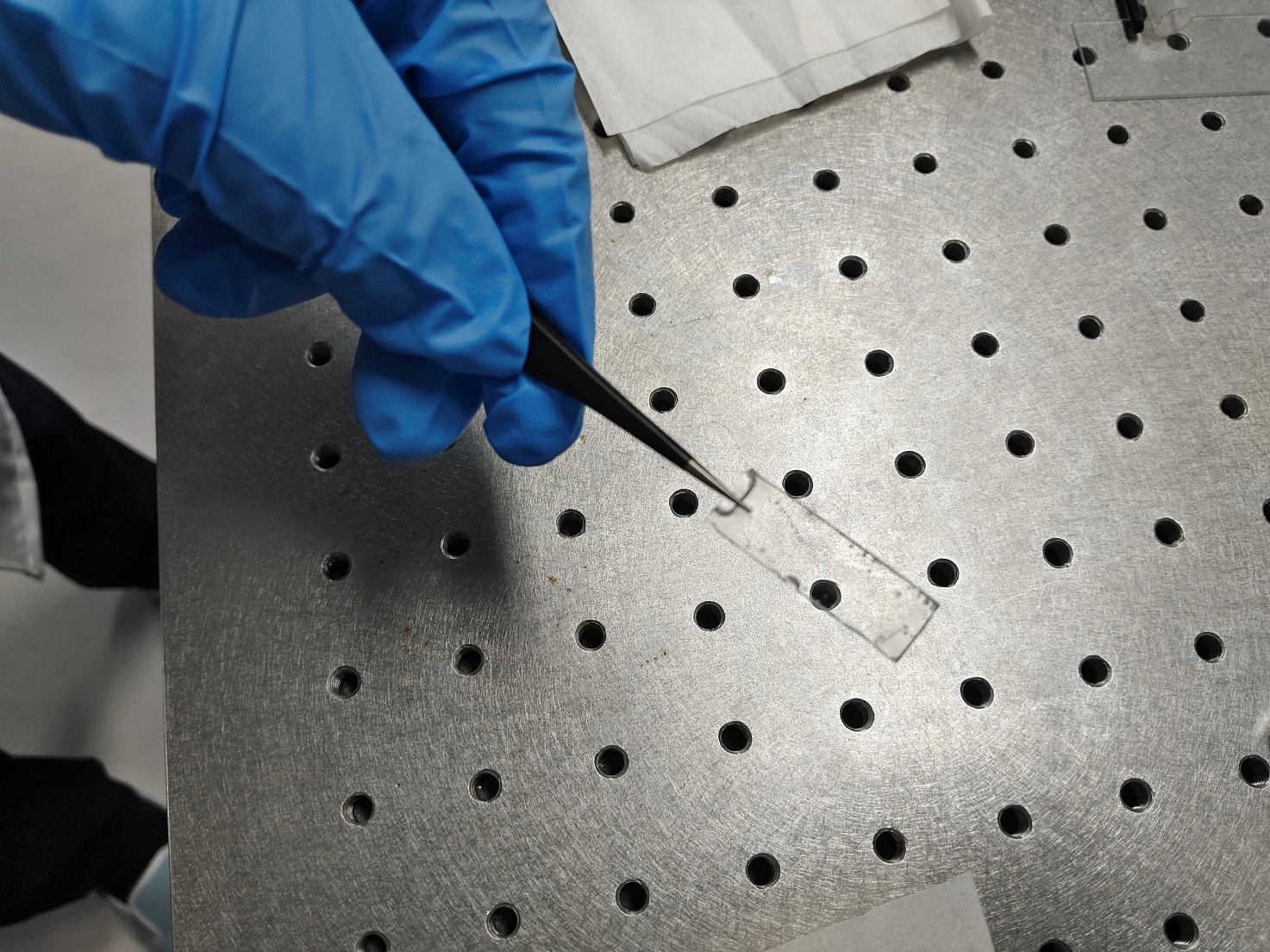


**Supplementary Figure 3**. Illustration of the sample prepared for the experiment.


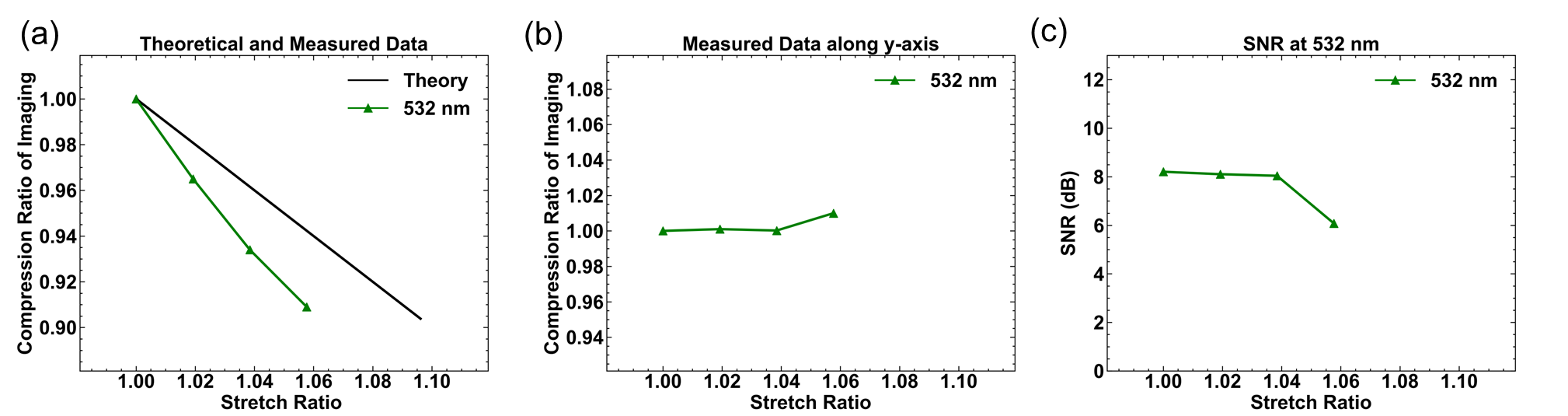


**Supplementary Figure 4**. Experimentally measured stretch ratio and signal to noise ratio at 532 nm. (a) The black solid line represents the strain ratio derived from the micrometer screw, while the colored solid lines represent the strain ratio derived from the diffraction order displacement. (b) The strain ratio derived from the diffraction order displacement in the *y*-direction during the experiment. (c) The experimentally measured signal-to-noise ratio.


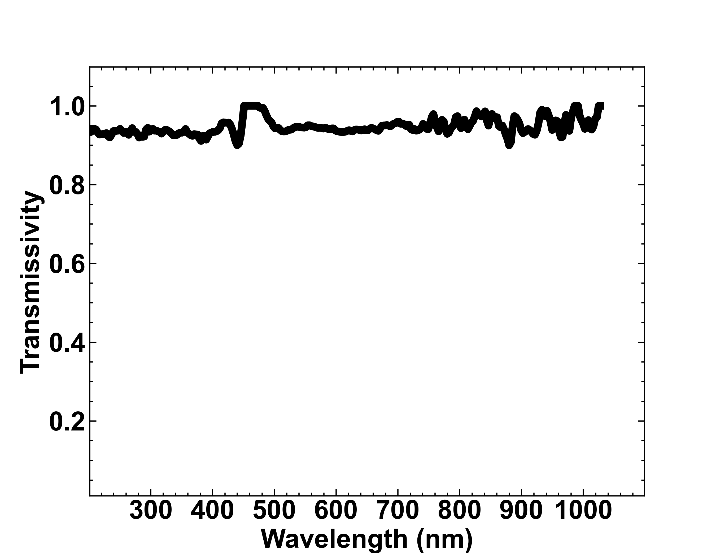


**Supplementary Figure 5**. Experimentally measured the transmissivity of PDMS in the visible to near-infrared range.


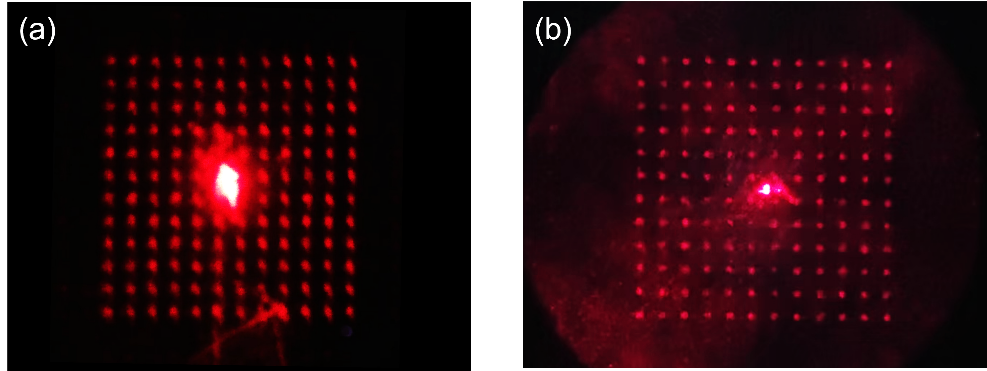


**Supplementary Figure 6**. The images obtained by the CCD in the reflection-type optical setup (a) and the transmission-type optical setup (b).

**Supplementary Note 1.**

It is noted that the signal-to-noise ratio decreases rapidly at a stretching ratio of 6% when the incident light is 532 nm, and it is submerged in speckle noise at approximately 8%. Hence, the normal operating range of the device at a wavelength of 532 nm is approximately 0% to 6%. Although geometric phase-type metasurfaces can generate signals under different incident wavelengths as a wide-band response metasurface, the actual sample’s signal-to-noise ratio and operating range depend not only on the plasmonic effects of the metasurface nanostructures and the sample’s processing precision but also on the scattering capability of the PDMS flexible material for different incident wavelengths under various stretching conditions. This is also one of the significant reasons for the differences in data obtained under different incident wavelengths and stretching ratios. Despite the experimentally measured strain ratio at an incident wavelength of 532 nm is larger than the theoretically predicted value, the discrepancy between the experimental and theoretical results does not exceed 3%. This still demonstrates the high reliability of the experimental results and the theoretical model, indicating that the theoretical model used can adequately describe the actual situation.
